# An Efficient Method of Key-Frame Extraction Based on a Cluster Algorithm

**DOI:** 10.2478/hukin-2013-0063

**Published:** 2013-12-31

**Authors:** Qiang Zhang, Shao-Pei Yu, Dong-Sheng Zhou, Xiao-Peng Wei

**Affiliations:** 1 Key Laboratory of Advanced Design and Intelligent Computing (Dalian University), Ministry of Education, Dalian, China.

**Keywords:** Motion Capture, ISODATA, Adaptive Threshold

## Abstract

This paper proposes a novel method of key-frame extraction for use with motion capture data. This method is based on an unsupervised cluster algorithm. First, the motion sequence is clustered into two classes by the similarity distance of the adjacent frames so that the thresholds needed in the next step can be determined adaptively. Second, a dynamic cluster algorithm called ISODATA is used to cluster all the frames and the frames nearest to the center of each class are automatically extracted as key-frames of the sequence. Unlike many other clustering techniques, the present improved cluster algorithm can automatically address different motion types without any need for specified parameters from users. The proposed method is capable of summarizing motion capture data reliably and efficiently. The present work also provides a meaningful comparison between the results of the proposed key-frame extraction technique and other previous methods. These results are evaluated in terms of metrics that measure reconstructed motion and the mean absolute error value, which are derived from the reconstructed data and the original data.

## Introduction

As the degree of verisimilitude that can be obtained from motion capture techniques increases, the significance and applicability of motion capture have also increased. Motion capture techniques have been widely used in computer animation, filmmaking, and gaming. Due to the tremendous volume of motion capture data, it is very important to store the data effectively and allow convenient browsing. This issue is similar to those raised by high-dimensional data, such as image and video information. The need for more compact representation has led researchers to investigate the ways of handling these large amounts of data.

Another problem with motion capture is editing the sequences so that the data can be reused. In motion capture, the animators can manufacture specific animation using the motion data collected from motion capture. However, if an animator wishes to modify only one part of the motion, he or she must modify the joint information from every frame, what is quite time-consuming.

If some key-frames capable of representing the motion captured can be extracted, then animators can more easily control the whole motion. Several attempts have already been made in this direction. The MoCaToon project focused on key-frames for generating animation (Morishima et al., 2007). Other projects have concentrated on editing, classifying, indexing, and compressing the motion capture data (Tilmanne et al., 2009; Clifford and Baciu, 2005; Baak et al., 2008; Bulut and Capin, 2007). Key-framing is one of the most effective ways to achieve the aim of editing and reuse of the motion capture data. The important frames of a motion can be selected for use as key-frames. There are many approaches to the selection of an appropriate frame for this purpose.

This paper focuses on two main aspects: first, an automatic key-frame extraction technique was developed based on the adaptive threshold. The adjacent frames were clustered in a motion sequence in order to identify the thresholds needed in the second step. A modified IOSDATA algorithm was used to identify the right key-frames. Second, a meaningful methodology was developed to compare results to those of two previous methods using reconstructed motion and the mean absolute errors from the reconstructed data and original data.

The rest of this paper is organized as follows: section 2 introduces some related works. Section 3 describes the present approach and provides some examples. Section 4 states the results of the approach and compares them with earlier ones. Finally, the conclusion and future works are discussed.

## Related Research

Key-frame extraction techniques have been used extensively in video retrieval, which is used for video browsing and content-based retrieval applications. Similarly, in recent years, various solutions to key-frame extraction have been proposed for motion capture data. These key-frame extraction methods can be divided into the following three categories: curve simplification, clustering, and matrix factorization based on the domain of the key-frame extraction problem.

### 

#### Curve simplification

The initial work of curve simplification was performed by Lim and Thalmann (Lim and Thalmann, 2001). They treated the motion sequence as a trajectory curve in the high-dimensional feature space and applied Lowe’s curve simplification algorithm to the extraction of key-frames according to a performer’s pose changes (Lowe, 1987). The extracted key-frames are the junctions between simplified curve segments. Li et al. (2005) together with Togawa and Okuda (2005) as well as Xiao et al. (2006) applied a novel layered curve simplification algorithm for motion capture data. Clifford and Baciu (2005) together with Halit and Capin (2011) used curve saliency for motion curves to specify the important frames of the motion (Clifford and Baciu, 2005; Halit and Capin, 2011). The obvious difference between these methods is the type of source data.

#### Clustering

Key-frame extraction becomes a clustering problem that attempts to group frames with similar posture. Liu et al. (2003) performed adaptive clustering-based key-frame extraction. A similarity measure between two frames was defined. They assigned each frame to a corresponding cluster by using the defined similarity. Park and Shin (2004) used quaternions to represent motion data and used PCA and k-means clustering to linearize quaternions and cluster the data. Then they used scattered data interpolation to extract key-frames from clustered motion data. More recently, Phillips et al. (2008) proposed a method of overlapping clustering based on network structure analysis. Takaki et al. (2007) used an underlying fuzzy clustering algorithm within the IGSCR framework to study the effects of using the fuzzy clustering algorithm. They all produced good results.

#### Matrix factorization

In this category, an animated sequence was represented as a matrix formed by placing all the vertices of a frame in a row (Huang et al., 2005). This matrix was then approximately factorized into a weight matrix and a key-frame matrix. Then the summary of motion was constructed using techniques such as singular value decomposition (SVD) and low-order discrete cosine terms (DCT) (Gong and Liu, 2000; Cooper and Foote, 2002).

These algorithms require a pre-specified experimental threshold, but these thresholds are not valid for all experimental data. Analyzing a movement without knowledge of the content of the motion is a very difficult problem with respect to the designation of experimental values that can influence the number of clusters. Incorrect initial values have considerable impact on experimental results. Different lengths of the movement sequences and differences in the type of motion render it impossible to set a uniform threshold for all experimental data and so produce good results.

On this basis, a new method of improving the above approach is proposed in the present paper. This algorithm belongs to the second method. The adjacent frames are clustered into two categories in order to determine the thresholds needed in the second step. Then, the improved IOSDATA algorithm is used to identify the correct key-frames.

## Approach to Key-Frame Extraction

The present approach consists of two main steps. In the first step, the similarity distances of the adjacent frames are used to cluster the motion sequence into two classes so that the thresholds needed for the clustering process of the second step can be determined adaptively. In the second step, a dynamic cluster ISODATA algorithm is used to cluster all the frames. Then, the frames nearest to the center of their class are automatically extracted for use as key-frames in the sequence.

ISODATA is one of the most popular clustering methods and is widely used in image processing in the geosciences and remote sensing applications. Wu et al. (2011) used modified ISODATA clustering to identify incorrect data identification regarding an integrated power system in warships. Memarsadeghi et al. (2007) introduced a more efficient approach to ISODATA clustering, which achieved better running times. Therefore, changes are still needed before either method can be applied to motion capture data.

### Division of the initial class and determination of the threshold

The human body is a joint chain structure, and its state can be expressed by the root joint translation and rotation of all the joints. In three-dimensional space, translation is usually a general three-dimensional vector, and rotation can be represented by a rotation matrix, Euler angles, or quaternions. Here Euler angles were used to represent the rotation. The similarity measure between two frames was defined using Euler’s formula:

(1) $$\mathrm{sim}\left(F_{i},F_{j}\right)=\sum_{t=1}^{n}\omega_{t}\sqrt{{\left(F_{i,t}-F_{j,t}\right)}^{2}}$$

where

*ω_t_* (i=1, 2… n) is the weighting factor, representing the importance of every joint. Because the human body can be considered a tree of joint chains, the movement of the child joint will be led by that of the parent joint, making the parent joint important to motion capture data. *n* is the number of joints in this motion capture scenario. *F_i_* represents the *i^th^* frame and *F_j_* represents the *j^th^* frame. *F_i,t_* represents the Euler angles that correspond to the *t^th^* joint of the *i^th^* frame, and *F_j,t_* is similar.

The approaches based on the similarity distance between two frames must involve specific experimental thresholds. Here, changes are made to the frames in order to render it more suitable for motion capture data. The steps include the following:

1. Let *F= {F_1_, F_2_, F_3_… F_N_}* denote the set of frames to be clustered, and use formula (1) to determine the similarity of two adjacent frames: *Dif* = {*D*_1_, *D*_2_,..., *D_N_*_−1_}.
2. Take the data in *Dif* as a set of sample to cluster and divide into two categories. *T* expresses the index in *Dif* when it is clustered. The formula for the calculation of dispersion of the two classes is as follows:
  (2) $$\begin{array}{l} \delta_{1}^{2}=\frac{1}{T}\sqrt{\sum_{i=1}^{T}\left(D_{i}-\text{arg}\left(\sum_{i=1}^{T}D_{i}\right)\right)}\\ \delta_{2}^{2}=\frac{1}{N-1-T}\sqrt{\sum_{i=T+1}^{N-1}\left(D_{i}-\text{arg}\left(\sum_{i=T+1}^{N-1}D_{i}\right)\right)}\\ \delta^{2}=\text{min}\left(\delta_{1}^{2}+\delta_{2}^{2}\right) \end{array}$$
  δ^2^ is the sum of dispersion. The set is divided into two categories when δ^2^ is minimum. Here, the current *D_T_* is the desired threshold.
3. If the similarity ≧ *D_T_*, go to another category. If the similarity ≧ *D_T_* between the current frame and the center of the current category, begin a new category.
4. If the algorithm stops, determine the number of categories and division of the initial classes.

This algorithm was used to test a large number of motion sequences. Due to the limited space, only the curve of walking is given here (418 frames). As shown in Picture 1, the abscissa of the curve is *Dif* in descending order and the ordinate is the corresponding δ^2^ when there are two categories of clusters in the *D_i_*. The abscissa of lowest point in the curve is 36. This indicates that the dispersion is minimum and *D_36_* is the required threshold. In addition, there are 35 elements greater than *D_36_*, so the number of initial classes is 37.

### Dynamic clustering and extraction of key-frames

After determining the number of categories and division of the initial class, a classical cluster algorithm, the ISODATA algorithm for dynamic clustering, was applied to the motion sequence. That is the 2nd clustering. The algorithm is not only capable of complete cluster analysis by adjusting the categories, but it can also automatically merge and split to obtain rational clustering. The ISODATA algorithm has some parameters to be set up, and the previous method artificially set the value of the parameters based on a priori knowledge of experimental data. The parameters corresponding to different data are different. Here, changes were made to the parameters to allow the system to adapt to the characteristics of human motion capture data and to reach the adaptive threshold. The algorithm is presented in more detail below. There are a number of user-supplied parameters (Wu et al., 2011; Memarsadeghi et al., 2007).

*K*: Expect the maximum number of clusters. If the sequence is very short, there is no need to extract a key-frame. *K* can be changed to adapt to the present reality. For example, the frame rate of the experimental data used here is 120 Hz, so the value of K is as follows:

(3) K=⌊N/25⌋

O*_N_*: Minimum number of frames that can form a cluster. *O_N_* can be changed to adapt to the actual situation. *O_N_* =25 would correspond to the value of *K* given above.

O*_S_*: Maximum standard deviation of points from their cluster center along each axis. Take the absolute value of difference of the various joints between the two frames (*F_i_* and *F_i+1_*) corresponding to *D_T_*. Here, the *O_S_* is dynamically set according to the new classification after splitting or merging such that the following is true:

(4) $$O_{s}=\sum_{t=1}^{n}\left|F_{i,t}-F_{i+1,t}\right|$$

O*_C_*: Minimum required distance between two cluster centers, value of *D_T_* in the first step. If the two frames (*F_i_* and *F_i+1_*) corresponding to *D_T_*, then this value must also be changed dynamically after splitting or merging again.

(5) $$D_{T}=\sum_{t=1}^{n}\omega_{t}\sqrt{{\left(F_{i,t}-F_{i+1,t}\right)}^{2}}$$

*L*: Maximum number of cluster pairs that can be merged per iteration. In this paper, adjacent frames are dealt with by using the rule of splitting or merging according to the order of the frames.

*I*: Maximum number of iterations. The classification number after each iteration is at the most half the number of the last classification. In this way, after a large number of experiments, the adequate number of iterations for certain motion capture data can be obtained.

(6) I=⌊N/(2*K)⌋

**Figure 1:**
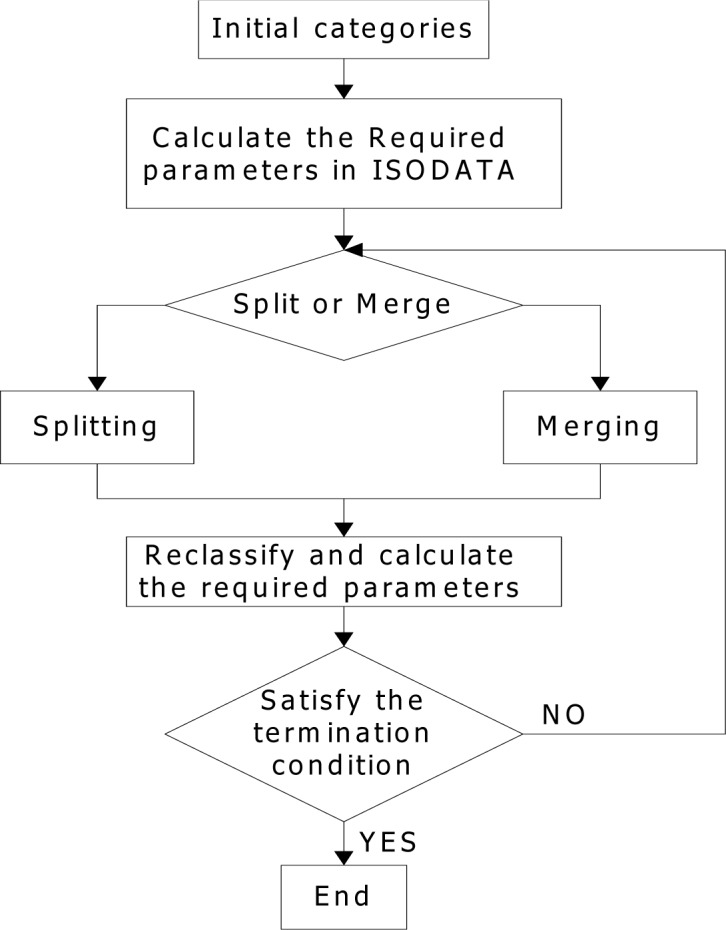
Proposed key-frame extractor

The values of *O_N_* and *L* are independent of the motion types and can take the same values in different motion sequences. However, the values of *K*, *O_S_*, *O_C_*, and *I* are obtained from the current movement. The method described above ensures that the thresholds are adaptively set to avoid the artificial setting. A flowchart of the proposed key-frame extraction algorithm is provided in Figure 1.

The following rules are used to split and merge the data:

1. Class splitting: If the dispersion within a certain class is greater than the mean dispersion δ̅ of various classes, and its maximum standard deviation is also greater than *O_S_*, split the class into two classes. If the classification number is less than *K/2* or if the number of iterations is odd and the classification number is between *K/2* and 2*K, then go to split.
2. Class merging: If the similarity measure of the centers of adjacent two categories is less than *O_C_* or the number of a certain class is less than *O_N_*, merge the two categories into one category. Again, if the classification number is greater than 2*K or if the number of iterations is even and the classification number is between *K/2* and 2*K, then go to merge.

When the expected number of clusters is achieved or the number of iterations reaches the maximum limit for the number of iterations *I*, end the cycle. After the final cluster, extract the frames closest to the centers of current categories for use as the key-frames of the motion sequence.

## Results

More than 100 real human motion sequences of different motion types were captured at a frame rate of 120 Hz from CMU as our testing collection and our method was implemented in Matlab® which runs on a Core(TM) 2 2.4 GHz computer with 4G memory (http://mocap.cs.cmu.edu).

At first, the algorithm was tested on three different types of motion: running, jumping, kicking a ball, swordplay, and playing. The parameters of each motion sequence are shown in Table 1. This method can meet the needs of key-frame selection in many motion sequences.

**Figure 2:**
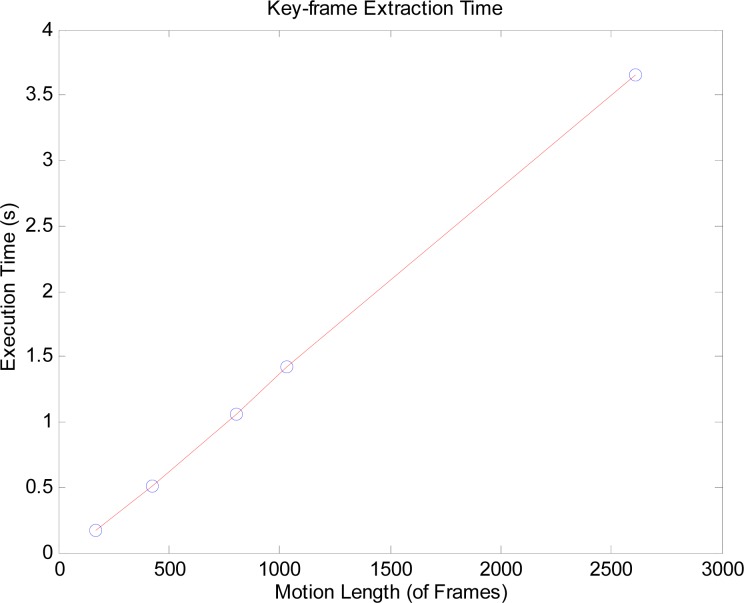
Key-frame extraction time of the present method for frame sequences of different lengths

Figure 2 displays the running times for different size of key-frames. As shown in the Figure above, for a motion of more than 2600 frames according to Table 1, the process takes less than 4 seconds. This shows that the running time of this approach increases along with the number of frames in a linear fashion. It can be concluded that the method can also be used effectively in real-time solutions.

Different methods can be applied: our approach, uniform sampling, and curve saliency extracting 8 key-frames from one motion sequence running (166 frames) (Bulut and Capin, 2007). The key-frames corresponding to the three different techniques are shown in Picture 2.

**Figure 3:**
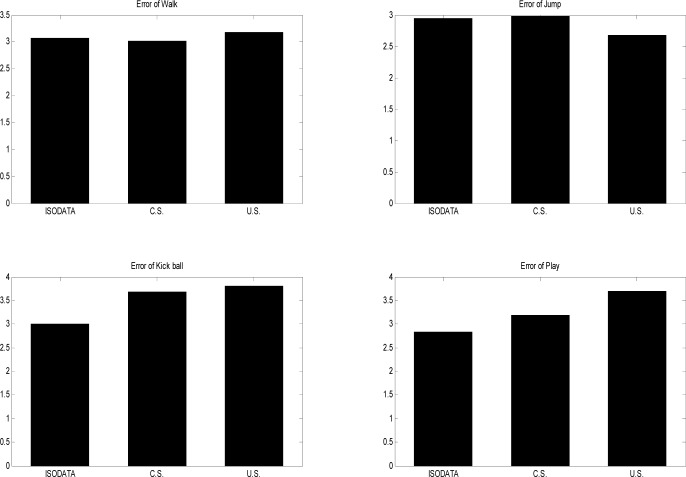
Comparison of mean absolute errors of four different types of motion using three different methods: ISODATA, curve saliency (C.S.), and uniform sampling (U.S.)

In Picture 2 (a)–(c), one periodic motion, running, is used to make a comparison between the different methods. For the same compression ratio the present approach achieves the best results, and the results produced using uniform sampling are better than those produced using curve saliency. The actual errors of reconstruction appear in the following Figure 3.

The three different methods given above were applied to four different types of motion types: walking, jumping, kicking a ball, and playing. Here, we also use the following method to compute the mean absolute error of our algorithm. The formula goes as follows:

(7) $$E=\text{log}10\left(\left[\sum {\left(Q(n)-Q'(n)\right)}^{2}\right]/N\right)$$

where

Q(n) and Q′(n) are the values of the joint in the original data and reconstructed data of motion, respectively. And when we expect the same number of key-frames in one motion type, we obtain the mean absolute error ratio as following in Figure 3.

As shown in Figure 3, three different methods are used to obtain the same number of key-frames from the same motion types. Then, a sequence of the motion is reconstructed in linear interpolation. The present approach is even worse than uniform sampling or curve saliency in (a) and (b). This is because the motion is too regular and only a small amount of information of some individual joints can reflect the changes in the whole movement in periodic motion, such as normal walking or running. However, the present method produces the expected results, which are better than uniform sampling and curve saliency in (c) and (d). They show that the performance of the present approach has more obvious advantages when applied to a motion with more irregular posture. Because the present algorithm involves the selection of key-frames from the original data, requiring no conversion of the motion data, it is not as time-consuming as other methods. It also works well with movement sequences involving large amounts of data, as shown in the present tests.

## Conclusion and Future Work

The main contribution of this paper is as follows: a modified method-based cluster algorithm, ISODATA, is proposed here for key-frame extraction from motion capture sequences. The sample is clustered into two categories using similarity distances of the adjacent frames in a motion sequence to produce the thresholds needed for the second step of the clustering process. Then, the improved ISODATA can be used for dynamic clustering and selection of the frames closest to the center of the final clustering for use as key-frames. A meaningful methodology for comparison with two previous methods was developed. This methodology involved reconstructed motion and the mean absolute error, both of which were from the reconstructed data and the original data.

Improvements to ISODATA clustering may allow this system to be used to cluster motion capture data to work with several different features, such as very regular movement. The running time can be further reduced. As shown in the results, although the present method also worked well with the motion sequences including different types of motion, its advantage is not obvious when it is used with very regular movements. In fact, previous methods outperform it in these cases. Rather, the present method has obvious advantages with movement sequences that involve irregular posture. The present approach can also facilitate good compression, which refers here to the relative number of key-frames to the total number of frames in the sequence.

## Figures and Tables

**Picture 1:**
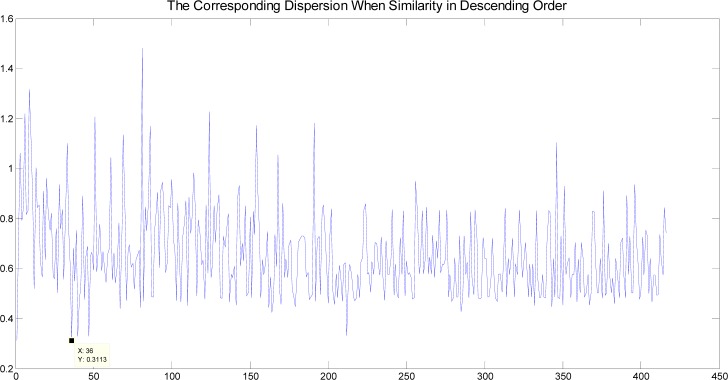
δ^2^ curve of a walking sequence

**Picture 2:**
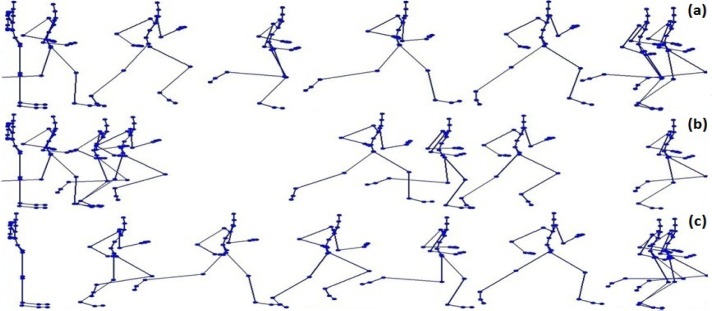
(a) ISODATA-based method. (b)Curve-saliency-based method; (c) Uniform-**sampling-based method**

**Table 1:** Key-frames for different types of motion

**Motion**	**Total frames**	**Key-frames**	**Compression ratio**	**Time (s)**	**Error ratio**
Running	166	8	4.2%	0.1672	3.7845
Jumping	427	19	4.4%	0.5086	2.9491
Kicking a ball	802	34	4.2%	1.0620	2.9998
Swordplay	1034	43	4.1%	1.4253	2.8348
Playing	2611	80	3.1%	3.6557	3.3482
